# The launch of prevention reporting: the 2017 national and federal state level workshop of German Federal Health Reporting

**DOI:** 10.17886/RKI-GBE-2018-049

**Published:** 2018-05-16

**Authors:** Susanne Jordan, Thomas Ziese, Ursula von Rüden

**Affiliations:** 1 Robert Koch Institute, Berlin, Department of Epidemiology and Health Monitoring; 2 Federal Centre for Health Education, Unit 2-25: Research, Quality Assurance, Cologne

The Robert Koch Institute (RKI) and the Federal Centre for Health Education (BZgA) organised a two-day workshop together with representatives from health reporting at the federal state level. The workshop, which took place between 9 and 10 November 2017 in Berlin, focused on two issues: ‘prevention reporting’ and ‘data sources’. The aim of the workshop was to share experiences about current developments and projects that are intended to implement prevention reporting at the federal and state level. A joint workshop on this topic was also conducted in 2016. The 2016 workshop focused on the methodological foundations of prevention reporting and relevant approaches. The 2017 workshop used the conclusions from 2016 to focus on the National Prevention Conference’s (NPC) Prevention Report and projects in prevention reporting that have already been implemented or that are planned at the federal state level.

The draft concept behind the NPC’s first prevention report, which will be prepared by the IGES Institute for the NPC, was presented by representatives of the institutions involved in the National Prevention Conference (Dr Liedtke, National Association of Statutory Health Insurance Funds; Dr Gravemeyer, German Social Accident Insurance; Dr Kamga Wambo, German Statutory Pension Insurance Scheme). They pointed out that the report will not only include data on the prevention measures being put in place by the NPC and information about the latest research on measures in particular settings (setting-based approach), but also data from the federal health monitoring and information from the federal state level. Ms Starker then explained the approach for the expertise of the RKI for the first NPK prevention report. She pointed out that the RKI expertise will bring together different data on the target groups of the Federal Framework Recommendations. This presentation was followed by three representatives from the federal state level who spoke about their state’s plans to implement prevention reporting. In Brandenburg, data on people’s health and social situation are being linked in the sense of integrated reporting; the results are to be used to develop proposals for targeted interventions (Ms Weigelt-Book, Brandenburg Ministry of Labour, Social Affairs, Health, Women and Family). In Baden-Württemberg, prevention reporting is seen as a form of research that accompanies the implementation of the Federal State Framework Agreement of Baden-Württemberg (which was introduced to map developments at the federal state and local level). As such, prevention reporting in Baden-Württemberg concentrates on existing structures (Dr Würz, Baden-Württemberg Ministry of Social Affairs and Integration). The presentation on the prevention indicators that are being applied in Bavaria made it clear that a prevention report can only ever focus on a selection of possible indicators. Furthermore, these indicators should ideally be updateable and readily available at different regional levels (Dr Reisig, Bavarian Health and Food Safety Authority).

During the workshop, the issue of prevention reporting was supplemented with information about new data sources and projects that are being undertaken at the RKI. Dr Gößwald provided a presentation about the next RKI examination survey of the adult population in Germany – field work is expected to begin in spring 2019. Ms Bartig provided a report about IMIRA (Improving Health Monitoring in Migrant Populations), a project aimed at improving the integration of migrants into the health monitoring conducted at the RKI. Dr Prütz outlined further RKI projects, such as the quarterly Journal of Health Monitoring, which the RKI edits and publishes, and three projects that deal with women’s health and gender equitable health reporting (Women’s Health Report; Women 5.0, and AdvanceGender). Dr Schmidt explained the tasks of diabetes surveillance at the RKI, and Mr Rommel described the activities and reforms that are taking place within the context of the Data Transparency Regulations (DaTraV). Finally, Dr von der Lippe used current data from the German Health Update (GEDA 2014/2015-EHIS) to demonstrate that differentiated evaluations are possible at the regional, federal state and European level.

The panel discussion with Dr Borrmann (North Rhine-Westphalia’s Centre for Health), Professor Geene (Magdeburg-Stendal University of Applied Sciences), Dr Kuhn (Bavarian Health and Food Safety Authority), Dr Starke (Academy of Public Health Services) and Dr Würz (Baden-Württemberg Ministry of Social Affairs and Integration) discussed fundamental questions and challenges associated with preparing a (national) prevention report. It concluded that prevention reports should provide insights that support disease prevention and health promotion in the institutions conducting the work on the ground (in other words, they should offer a practical orientation). The debate demonstrated that it was important not to place too many hopes either on the first report – which is due to be published in 2019 – or on those that follow in terms of immediately quantifiable impacts, since preventive interventions and health promotion measures are complex and involve elaborate evaluations and different methodological requirements. Moreover, most of the measures that have been planned have yet to be implemented; as such, no measurable effects can be expected before 2019. At the same time, the focus of the Preventive Health Care Act means that it is important to ensure that cooperative and participatory structures at the municipal level and small-scale evaluations and needs analyses are taken into account. The paradigm shift that is taking place in prevention and health promotion is characterised by measures taken in people’s living environments (‘settings approach’), resource promotion, structural prevention and health-in-all-policies strategies. These changes will also need to be reflected in prevention reporting in the future. However, doing so will also require additional indicators. In the long term, an assessment will be needed of how a common set of core indicators could be designed so that they can be applied by all departments – and at the state, federal, and, if possible, local level. These indicators would also have to take current key requirements and developments in prevention and health promotion into account.

The workshop highlighted the fact that the Preventive Health Care Act is already providing important impulses at the federal and state level for the conceptual and practical implementation of prevention and intervention reporting. Some federal states have developed concepts or begun implementing initial projects in prevention reporting. This was also clear from the answers provided by the representatives present at the workshop and from the lectures presented by the federal states. These developments provide an opportunity to strengthen prevention and health promotion in Germany by using advances in knowledge and evidence-based approaches.

